# Association between p62 expression and clinicopathological characteristics in oral leukoplakia

**DOI:** 10.1002/cre2.193

**Published:** 2019-06-25

**Authors:** Toshio Yoshida, Takehito Terabe, Hiroki Nagai, Fumihiko Uchida, Shogo Hasegawa, Toru Nagao, Satoru Miyabe, Naomi Ishibashi‐Kanno, Kenji Yamagata, Eiji Warabi, Masahiko Gosho, Toru Yanagawa, Hiroki Bukawa

**Affiliations:** ^1^ Oral and Maxillofacial Surgery, Clinical Sciences, Graduate School of Comprehensive Human Science University of Tsukuba Tsukuba Japan; ^2^ Yoshida Dental Office Medical Cooperation Tokuekai Ishioka Japan; ^3^ Department of Oral and Maxillofacial Surgery, Association for Development of Community Medicine Ishioka Daiichi Hospital Ishioka Japan; ^4^ Department of Oral and Maxillofacial Surgery, Faculty of Medicine University of Tsukuba Tsukuba Japan; ^5^ Department of Maxillofacial Surgery, School of Dentistry Aichi‐Gakuen University Nagoya Japan; ^6^ Department of Oral and Maxillofacial Surgery Toyota Wakatake Hospital Toyota Japan; ^7^ Department of Anatomy and Embryology, Faculty of Medicine University of Tsukuba Tsukuba Japan; ^8^ Department of Biostatistics, Faculty of Medicine University of Tsukuba Tsukuba Japan

**Keywords:** autophagy, epithelial dysplasia, oral leukoplakia, p62

## Abstract

**Objective:**

Oral leukoplakia is keratinized lesions in the buccal mucosa, tongue, and gingiva. It is the most common oral precancerous lesion; oxidative stresses and irrelevant autophagy have been reported to be the cause of oncogenesis. p62, a cytoplasmic protein induced by oxidative stress, is an adaptor protein involved in the formation of protein aggregates and induction and inhibition of autophagy. The inhibition of autophagy induces p62 overexpression and promotes oncogenesis via the oncogenic signaling pathway. The aim of the present study was to elucidate the involvement of intracellular expression of p62 in oral leukoplakia and to address its potential clinical implementation as a biomarker to predict malignant transformation.

**Material and Methods:**

Fifty samples from subjects with confirmed oral leukoplakia were evaluated by immunohistochemical staining for the expression of p62, 8‐hydroxy‐2′‐deoxyguanosine (8‐OHdG), Ki67, and p53. Univariate and multivariate logistic regression analyses were performed to evaluate the association between p62, 8‐OHdG, Ki67, and p53 and clinical characteristics, including epithelial dysplasia.

**Results:**

Significant associations were observed between p62 expression in the nucleus, p62 aggregation, and epithelial dysplasia (adjusted odds ratio [OR] = 5.75; 95% confidence interval [CI]: [1.28, 26.2]; .024 and OR = 6.16; 95% CI: [1.01, 37.4]; .048, respectively). The expression of p62 in the cytoplasm and the levels of 8‐OHdG, Ki67, and p53 were not significantly associated with epithelial dysplasia. A significant relationship was found between p62 expression in the nucleus and p53 expression (OR = 3.94; 95% CI: [1.14, 13.6]; .031).

**Conclusions:**

The results suggested that p62 expression in the nucleus and p62 aggregation can be potential markers to predict the malignant transformation of oral leukoplakia.

## 1 INTRODUCTION

Oral leukoplakia is keratinized lesions occurring in the buccal mucosa, tongue, and gingiva. Oral leukoplakia, especially in the tongue, has a high probability of transforming into malignancy (Bánóczy & Csiba, 1976). The potential for malignant transformation of oral leukoplakia ranges from 4.4% to 17.5% (Pindborg, Jølst, Renstrup, & Roed‐Petersen, 1968; Silverman, Gorsky, & Lozada, 1984). Oral leukoplakia is known to be the most common oral precancerous lesion classified as an oral potentially malignant disorder (Warnakulasuriya, Johnson, & van der Waal, 2007). As it is important to predict the possibility of malignant transformation and prognosis of oral leukoplakia, several attempts have been made to identify specific molecular biomarkers. Recently, surgical treatments underwent new considerations concerning the widespread use of anticoagulant drugs in cardiovascular disease and became high‐risk procedures (Isola et al., 2015).

Nagao et al. (2017) studied the expression of p53 and Ki67 in response to chemoprevention of oral leukoplakia and reported that the expression of p53 is inversely associated with the clinical response to supplements administered for the condition. Studies have also reported the biomarkers involved in oral leukoplakia (Nikitakis et al., 2018), such as cornulin, keratin 4, keratin 13 (Schaaij‐Visser et al., 2010), and podoplanin (de Vicente et al., 2013). Zamora et al. indicated that the salivary 8‐OHdG level increased in patients with chronic periodontitis and aggressive periodontitis. They also reported a positive correlation between oxidative stress and increased micronuclei in patients with periodontitis (Zamora‐Perez et al., 2015). This suggests the association between oxidative stress and leukoplakia. In a research on gene expression of malignant transformation, Ferlazzo et al. (2017) reported that a methylenetetrahydrofolate reductase polymorphism influenced DNA methylation and played an important role during oral carcinogenesis. Additionally, Chaves et al. (2019) reported that the levels of CD8^+^ cells increased in the oral premalignant region that later became oral squamous cell carcinoma.

p62 is an oxidative stress protein implicated in the prognosis and survival of various malignancies, including hepatocellular, breast, prostate, ovarian, some gastrointestinal carcinomas, and melanoma.(Ruan et al., 2017) Furthermore, it has been reported to be associated with autophagy. p62 has been cloned as A170/Sequestosome/ZIP, and its expression is induced by oxidative stress and other stresses. Previously, we cloned cDNA encoding a novel oxidative stress protein designated A170 from murine peritoneal macrophages (Ishii et al., 1996). The ubiquitin‐ and LC3‐binding protein p62 regulates the formation of protein aggregates, which is removed by autophagy. Therefore, the inhibition of autophagy induces a marked cytoplasmic expression of p62 (Komatsu et al., 2007). Recently, it has been reported that oncogenic signaling is mediated by the expression of p62, promoting the biogenesis and proliferation of cancer. Furthermore, recent studies have indicated abnormal overexpression of p62 in oral squamous carcinoma cells, and this might be the main cellular defense mechanism against cancer therapy (Inui et al., 2013). Overexpression of p62 was more obvious in oral carcinoma than in low‐grade dysplasia or nonepithelial dysplasia. Overexpression of p62 can cause low‐grade dysplasia in the oral epithelium, which can develop into carcinoma via resistance to various oxidative stresses (Inui et al., 2013). Histologically, epithelial dysplasia is indicated as one of the factors involved in the carcinogenesis of oral leukoplakia.

If it is proved that p62 expression and epithelial dysplasia are significantly correlated, this could lead to the implementation of p62 as a marker predicting malignant transformation that could be applied clinically, which would substantially improve the treatment and diagnosis of oral leukoplakia.

The aim of the present study was to elucidate the involvement of intracellular expression of p62 in oral leukoplakia. We evaluated the intracellular expression of p62 in oral leukoplakia tissue specimens and compared various clinical characteristics, such as the intracellular expression of p62 and dysplasia in the oral epithelium, as well as other biomarkers including 8‐hydroxy‐2′‐deoxyguanosine (8‐OHdG), Ki67, and p53 associated with oral leukoplakia.

## 2 MATERIAL AND METHODS

### 2.1 Samples

Fifty formalin‐fixed, paraffin‐embedded blocks prepared from specimens of Japanese Asian patients with pathologically diagnosed oral leukoplakia who visited four city hospitals (viz., Aichi‐Gakuen University School of Dentistry, Okazaki City Hospital, Aichi Saiseikai Hospital, and Yokkaichi Municipal Hospital) from 1991 to 2015 were retrospectively collected in Japan. Clinical information and clinicopathological data were obtained from medical records. Patients with oral leukoplakia included 24 males (48%) and 26 females (52%) with a median age of 68 (28–95 years). The clinical‐demographic characteristics of the study participants, along with data regarding the presence or absence of epithelial dysplasia, are presented in Table 1. The oral leukoplakia specimens were harvested from the tongue (18 cases; 36%) and other areas (32 cases; 64%); 24 of 50 patients (48%) were smokers (two patients were not examined), and 18 (36%) were habitual alcohol drinkers (one patient was not examined). The number of patients with epithelial dysplasia was 18 (36%). The number of patients with single occurrence lesions was 43 (86%), and the number of patients with multiple occurrence lesions was 7 (14%).

**Table 1:** Clinical characteristics (number of cases and %)

Characteristics	Number of cases (%)
Age	
≤64	20 (40)
≥65	30 (60)
Sex	
Male	24 (48)
Female	26 (52)
Location	
Tongue	18 (36)
Others	32 (64)
Occurrence form	
Single	43 (86)
Multiple	7 (14)
Drinking	
Yes	18 (36)
No	31 (62)
Not examined	1 (2)
Smoking	
Yes	24 (48)
No	24 (48)
Not examined	2 (4)
Epithelial dysplasia	
Positive	18 (36)
Negative	32 (64)

The relevant data were collected from clinical and pathological records after obtaining written consent from the patients and approval by the Ethics Committee of Aichi‐Gakuin Dental Hospital, Nagoya, Japan (approval number 39). This study was performed in compliance with the Declaration of Helsinki.

### 2.2 Immunohistochemical staining

#### 2.2.1 p62 staining

All the specimens were serially sectioned to a thickness of 4 μm. After deparaffinization, the tissue sections were pretreated with 10 mmol/L of sodium citrate buffer (pH 6.0) in a microwave oven for 5 min at 95°C. The sections were then incubated in a mixture of 0.3% hydrogen peroxide solution in 100% methanol for 20 min to quench endogenous peroxidase activity. After washing with phosphate‐buffered saline, the sections were incubated overnight at 4°C with the primary antibodies for p62 (1:100; SQSTM1, ab56416; Abcam, Cambridge, UK). After incubation, the sections were rinsed with phosphate‐buffered saline and treated with the secondary antibody (Vector Laboratories, Burlingame, CA) for 30 min, followed by color development using 3,3′‐diaminobenzidine tetrahydrochloride (DAB; Cell Signaling Technology, Tokyo, Japan) to detect antigen–antibody binding, and hematoxylin was applied for 1 min to counterstain the nuclei. Epithelial cells from the plantar of mice were used as positive control. The samples stained without the primary antibody were used as negative control.

#### 2.2.2 8‐OHdG staining

All the specimens were serially sectioned to a thickness of 4 μm. After deparaffinization, the tissue sections were pretreated with 10 mmol/L of sodium citrate buffer (pH 6.0) in a microwave oven for 20 min at 90°C. However, 0.3% hydrogen peroxide solution was not used because 8‐OHdG has a specific immune stainability for the nucleus. After washing with phosphate‐buffered saline, the sections were incubated overnight at 4°C for 6–12 hr with the primary antibody (1:200 anti‐8‐hydroxy‐2′‐deoxyguanosine antibody, cloneN45.1; JaICA, NIKKEN SEIL, Tokyo, Japan). After incubation, the sections were rinsed with phosphate‐buffered saline and treated with the secondary antibody (Vector Laboratories, Burlingame, CA) for 30 min, followed by color development using 3,3′‐diaminobenzidine tetrahydrochloride (DAB; Cell Signaling Technology, Tokyo, Japan) for 30 min to detect antigen–antibody binding, and hematoxylin was applied for 20 s to 1 min to counterstain the nuclei. Epithelial cells from the plantar of mice were used as positive control. After setting the conditions, immunohistochemical staining was performed using an automated immunostainer (Histostainer48A; Nichirei Biosciences Inc., Tokyo, Japan).

#### 2.2.3 Ki67 and p53 staining

Samples were immunostained for Ki67 (Dako monoclonalmouse Clone MIB‐1; Dako, Glostrup, Denmark) and p53 (Dako monoclonalmouse Clone DO‐7; Dako) using an automated immunostainer (Dako Autostainer Link; Dako) by the standard streptavidin‐biotin‐peroxidase method (Dako EnVision FLEX kit; Dako, Glostrup, Denmark). The antigen‐retrieval technique included high‐temperature heating in a microwave oven for 10 and 5 min for Ki67 and p53, respectively.

#### 2.2.4 Evaluation of expression of p62, 8‐OHdG, Ki67, and p53

**Figure 1:**
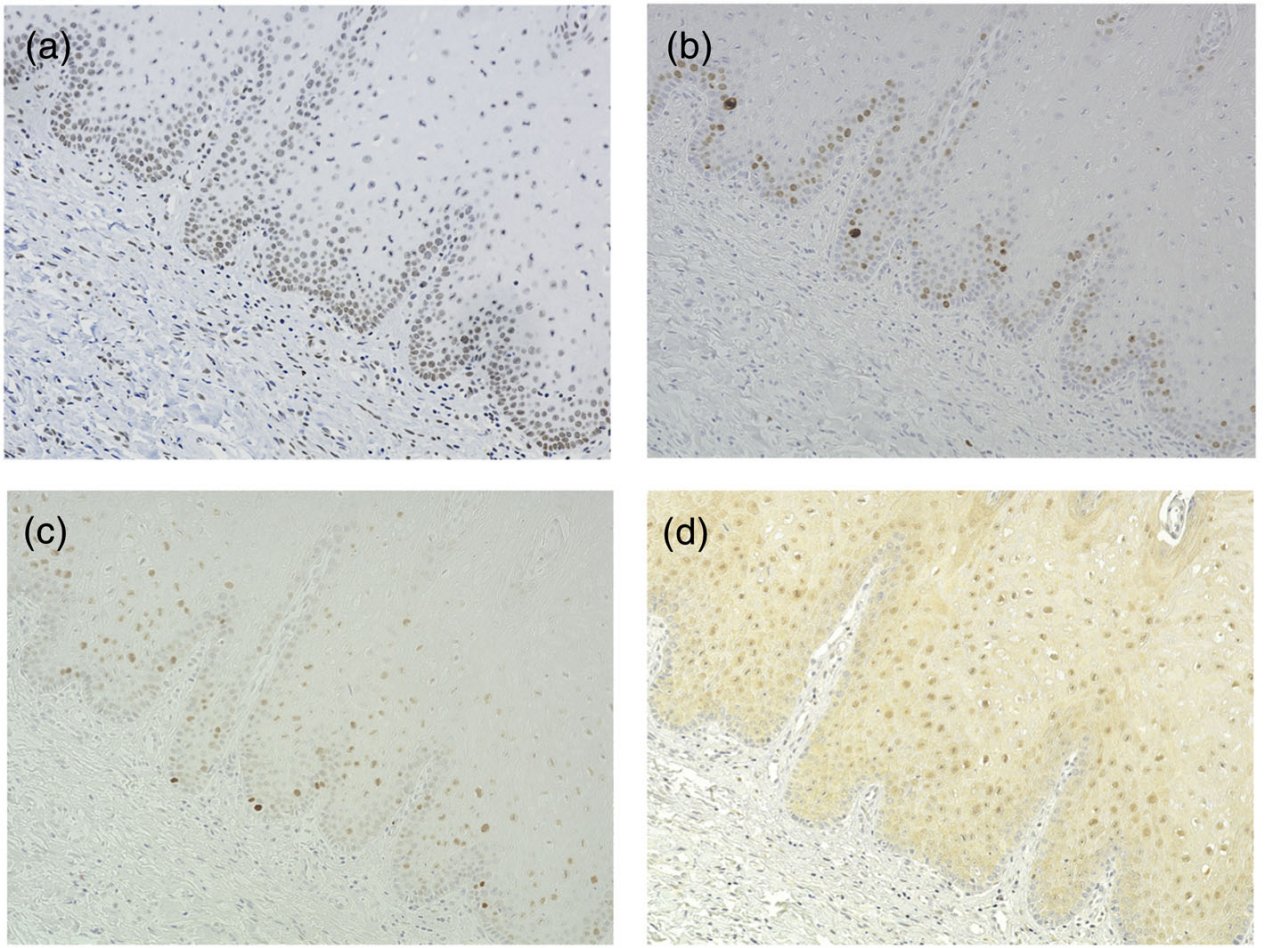
Immunohistochemical staining of (a) 8‐OHdG, (b) Ki67, (c) p53, and (d) p62 in oral leukoplakia tissue specimens. Representative sections showing positive expression of the four proteins. Magnification: 200×. (d) Section showing positive expression of p62 in the nucleus and cytoplasm

**Figure 2:**
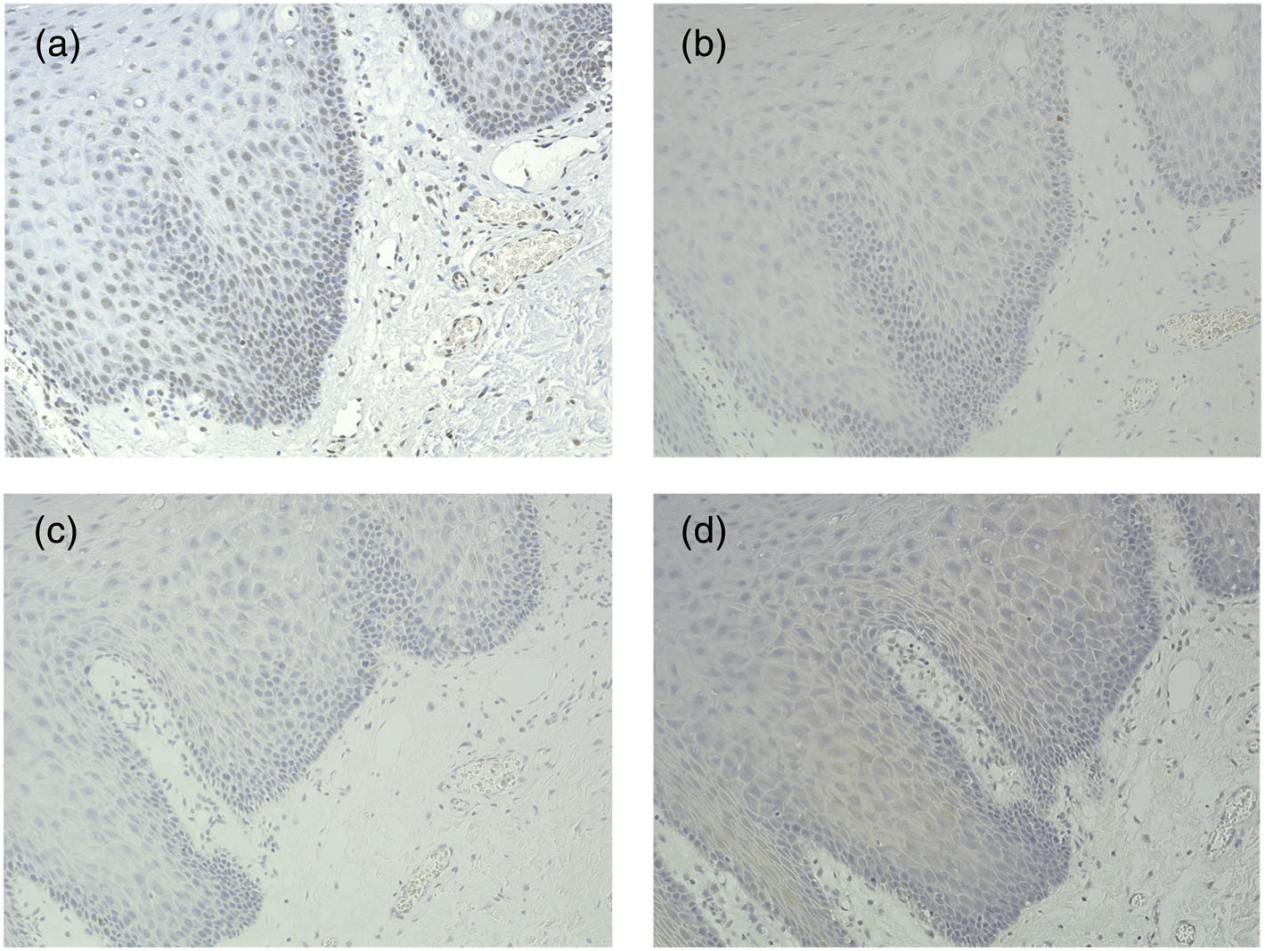
Immunohistochemical staining of (a) 8‐OHdG, (b) Ki67, (c) p53, and (d) p62 in oral leukoplakia tissue specimens. Representative sections showing negative expression of the four proteins. Magnification: 200×. (d) Section showing negative expression of p62 in the nucleus and cytoplasm

The stained sections were independently evaluated by one oral pathologist (S.M.), and three oral surgeons (T.Y., T.T., and H.N.) blindfolded. We evaluated the expression of p62, 8‐OHdG, and p53 in the mucosal epithelium and the expression of Ki67 in the basal or suprabasal layer. Although both strong and weak expressions were observed, we only judged the specimens as either positive or negative. Epithelial cells from the plantar of mice were used as positive control. Samples stained without the primary antibody were used as the negative control. Representative features are shown in Figures 1 and 2.

All immunohistochemical markers were assessed by light microscopy (BZ‐X700 All‐in‐One Fluorescence Microscope; KEYENCE, Osaka, Japan). The areas showing the expression of representative markers in the sections were chosen at a low‐powered field (100×), and then evaluated at middle‐ and high‐powered fields (200× and 400×, respectively). Three oral surgeons (T.Y., T.T., and H.N.) blindfolded and independently counted the positive cells for each marker and calculated the positive cell occupancy rate to identify the cutoff value, considering each receiver‐operating characteristic. We evaluated the markers in each sample at levels of 1%, 10%, 20%, 30%, and 50% positivity by the mean value of the positive cell occupancy rate and constructed the corresponding class values, histograms, box plots, and receiver operating characteristic curves. We differentiated nuclear and cytoplasmic expression of p62 because differences in p62 localization indicate other conditions (Komatsu et al., 2010). Positive expression of nuclear p62 was defined as p62 staining in the nuclei. A cutoff value of 20% was used to group cases with cells presenting positive expression of nuclear p62. With respect to p62 aggregation, ≥1 dot per cell in more than 1% of the oral leukoplakia tissue at 200× and 400× magnification fields was defined as positive expression, and other results were defined as negative expression. Thus, a cutoff value of 1% was used to group cases presenting p62 aggregation. Positive expression of cytoplasmic p62 was defined as p62 staining in the cytoplasm. A cutoff value of 20% was used to group cases with cells presenting positive expression of cytoplasmic p62. If 8‐OHdG staining was observed in the nuclei, we judged the cell as a positive expression cell. Positive expression of nuclear p53 was defined as nuclear positivity for p53 in the epithelium and positive expression of Ki67 was defined as nuclear positivity for Ki67 in the basal or suprabasal layer. A minimum of 1,000 cells were counted under 200× and 400× magnifications in areas showing the expression of representative markers in each section. Similarly, we used a cutoff values of 50%, 10%, and 1% for 8O‐HdG, Ki67, and p53 positive cells, respectively. Positive cell occupancy rate of each maker is shown in Figures S1 and S2.

### 2.3 Statistical analyses

We performed univariate and multivariate logistic regression analyses to evaluate the association between epithelial dysplasia and other clinical characteristics. We also performed a logistic regression analysis to estimate the association between epithelial dysplasia and each biomarker in oral leukoplakia tissue samples. We performed a univariate logistic regression analysis to assess the relationship between p62 expression and aggregation and other biomarkers. Results with *P* values <.05 were considered statistically significant. All statistical analyses were performed with JMP® 13 for WIN (SAS Institute Inc., Cary, NC, USA).

## 3 RESULTS

The clinicopathological characteristics including age, gender, location, occurrence type, drinking habit, smoking habit, and epithelial dysplasia are shown in Table 1. The relationship between epithelial dysplasia and clinical characteristics is shown in Table 2. The number of epithelial dysplasia (+) samples was as follows: in patients aged ≤64: 9 of 20 (45%), in those aged ≥65: 9 of 30 (30%); in males: 12 of 24 (50%), in females: 6 of 26 (23%); in the tongue region: 11 of 18 (61%), in other regions: 7 of 32 (22%); in patients with single occurrence: 14 of 43 (32%), in those with multiple occurrence: 4 of 7 (57%); in patients with drinking habit: 9 of 18 (50%), in those with no drinking habit: 9 of 32 (28%); in smokers: 11 of 24 (46%), in nonsmokers: 7 of 26 (27%). There was a significant association between epithelial dysplasia and location in the univariate logistic regression analysis (.046), but there was no significant relationship between epithelial dysplasia and other clinicopathological characteristics in the multivariate analysis (Table 2). The results confirmed that other clinical characteristics did not affect epithelial dysplasia (data not shown).

**Table 2:** Epithelial dysplasia and other clinical parameters

Characteristics	Odds ratio	95% CI	P value
Univariate logistic regression analysis			
Age			
≦64 vs. ≦65	1.91	[0.59, 6.20]	.282
Sex			
Male vs. female	3.33	[0.99, 11.22]	.052
Location			
Tongue vs. others	3.57	[1.03, 12.43]	.046*
Occurrence form			
Single vs. multiple	0.36	[0.07, 1.84]	.221
Drinking			
Yes vs. no	2.44	[0.73, 8.17]	.146
Smoking			
Yes vs. no	2.05	[0.62, 6.76]	.236
Multivariate logistic regression analysis			
Age			
≦64 vs ≦65	1.76	[0.43, 7.18]	.434
Sex			
Male vs. female	3.23	[0.43, 24.40]	.257
Location			
Tongue vs. others	2.45	[0.59, 10.20]	.220
Occurrence form			
Single vs. multiple	0.41	[0.06, 2.75]	.361
Drinking			
Yes vs. no	1.00	[0.18, 5.49]	.998
Smoking			
Yes vs. no	1.55	[0.19, 12.36]	.681

* *P* < 0.05 significant.

The relationships between epithelial dysplasia and each biomarker are shown in Table 3. With respect to the number of epithelial dysplasia (+) samples, the following was observed: nuclear p62 (−): 7 of 29 (24%); nuclear p62 (+): 11 of 21 (52%); p62 aggregation (−): 10 of 36 (28%); p62 aggregation (+): 8 of 14 (57%); cytoplasmic p62 (−): 7 of 21 (33%); cytoplasmic p62 (+): 11 of 29 (38%); 8‐OHdG (−): 4 of 18 (22%); 8‐OHdG (+): 14 of 32 (44%); Ki67 (−): 14 of 40 (35%); Ki67 (+): 4 of 10 (40%); p53 (−): 6 of 21 (29%); and p53 (+): 12 of 29 (41%). There was a significant association between epithelial dysplasia and p62 in the nucleus in the univariate logistic regression analysis (.044). Furthermore, there was a significant association between epithelial dysplasia and p62 in the nucleus (.024) and p62 aggregation (.048) in the multivariate analysis.

**Table 3:** Logistic regression analysis in association with dysplasia and each biomarker

Parameter	Odds ratio	95% CI	P value
Univariate logistic regression analysis			
p62 in nucleus	3.46	[1.02, 11.56]	.044*
p62 aggregation	3.47	[0.96, 12.54]	.058
p62 in cytoplasm	1.22	[0.38, 3.97]	.738
8‐OHdG	2.72	[0.73, 10.11]	.135
Ki67	1.24	[0.30, 5.13]	.769
p53	1.76	[0.53, 5.87]	.354
Multivariate logistic regression analysis			
p62 in nucleus	5.75	[1.26, 26.19]	.024*
p62 aggregation	6.16	[1.01, 37.42]	.048*
p62 in cytoplasm	1.90	[0.42, 8.62]	.403
8‐OHdG	5.31	[0.96, 29.46]	.056
Ki67	1.46	[0.27, 7.76]	.658
p53	2.35	[0.57, 9.76]	.240

* *P* < 0.05 significant.

The relationships between p62 parameters (p62 in the nucleus, p62 aggregation, and p62 in the cytoplasm) and other biomarkers (8‐OHdG, Ki67, and p53) are shown in Table 4. There was a significant association between p62 in the nucleus and p53 (.031). The results indicated that p62 expression in the nucleus and p62 aggregation were significantly associated with the occurrence of epithelial dysplasia.

**Table 4:** Univariate logistic regression analysis in association with p62 parameters and other biomarkers

Parameter	Odds ratio	95% CI	P value
p62 in nucleus			
8‐OHdG	2.60	[0.75, 9.01]	.132
Ki67	4.33	[0.97, 19.43]	.055
p53	3.94	[1.14, 13.65]	.031*
p62 aggregation			
8‐OHdG	1.59	[0.42, 6.07]	.497
Ki67	2.00	[0.47, 8.56]	.350
p53	3.67	[0.87, 15.38]	.076
p62 in cytoplasm			
8‐OHdG	1.67	[0.52, 5.36]	.392
Ki67	3.62	[0.68, 19.21]	.131
p53	2.09	[0.66, 6.59]	.354

* *P* < 0.05 significant.

## 4 DISCUSSION

In the present study, we found that the association between the intracellular expression of p62 and the presence of epithelial dysplasia and other clinical characteristics are involved in oral leukoplakia. The univariate analysis showed a significant association between epithelial dysplasia and location (*P* < .05), but the multivariate analysis showed no significant relationship between epithelial dysplasia and other clinicopathological characteristics (Table 2); these results confirmed that other clinical characteristics did not affect epithelial dysplasia. The univariate analysis showed a significant association between epithelial dysplasia and p62 in the nucleus (*P* < .05), and the multivariate analysis showed that the positive nuclear p62 expression and the positive p62 aggregation groups included the epithelial dysplasia group of oral leukoplakia (*P* < .05; Table 3). Considering these results and by removing the confounding factors, it was clarified that the aberrant expression of nuclear p62 and p62 aggregation affect the occurrence of epithelial dysplasia. The pathogenesis and epidemiological explanation of oral leukoplakia have been previously described (Leemans, Braakhuis, & Brakenhoff, 2011).

The oral mucosal epithelium is often exposed to various stresses, including tobacco, alcohol, periodontal disease, and other inflammations, salt, stimulant luxury goods, and other environmental and lifestyle stresses (Nagao et al., 2016; Petti, 2009). Several causal factors for oral leukoplakia are known; these include smoking, excess alcohol drinking, betel quid chewing, and chronic infections by fungi and viruses (Petti, 2009). It was reported that periodontitis increased the risk of oral leukoplakia and mucosal lesions, thus predisposing to oral cancers (Meisel, Holtfreter, Biffar, Suemnig, & Kocher, 2012).

Dental prosthesis and restorations have been reported in association with oral leukoplakia as risk factors in Japanese subjects (Nagao et al., 2016). It has been suggested that these stresses might be sources of massive quantities of reactive oxygen species, which have been clearly recognized as etiologic factors of precancerous progression, as damage to cells easily accumulates. These changes alter the oxidant–antioxidant status of various clinical stages of oral leukoplakia presumably (Srivastava, Austin, Shrivastava, & Pranavadhyani, 2014). If oxidative stress and other stresses accumulate in the oral mucosa, intracellular homeostasis might be altered, and autophagy might be impaired (Żukowski, Maciejczyk, & Waszkiel, 2018).

Kiffin, Bandyopadhyay, and Cuervo (2006) reported that mild oxidative stress promotes successful autophagy to facilitate the elimination of damaged organelles, but acute or persistent oxidative stress induces an intracellular increase in reactive oxygen species, which injure the lysosomal membrane and disturb the formation of autophagosomes. If autophagy is not functional or the oxidative stress damage exceeds autophagy, aberrant and altered intracellular oxidized components (including p62) are accumulated in cells. In oral leukoplakia lesions, a similar response to oxidative injury might occur as part of cellular defense systems (Kiffin et al., 2006). We can hypothesize another association between oxidative stress and autophagy. Under basal conditions, the Nrf2–Keap1 pathway functions as a critical regulator of cellular defense mechanisms against oxidative stress (Lau et al., 2010). p62 interacts with the Nrf2‐binding site on Keap1, and overexpression of p62 or autophagy deficiency results in a competition between Nrf2 and p62 for Keap1, resulting in the stabilization of Nrf2 and transcriptional activation of the antioxidant response element, including p62 (Komatsu et al., 2010). Such consecutive reactions break the loop, and p62 creates a positive feedback loop in the Keap1–Nrf2 pathway (Jain et al., 2010). This noncanonical mechanism of Nrf2 might explain p62 nuclear overexpression and p62 aggregation during oral leukoplakia under acute or persistent oxidative stress conditions.

In the present study, we speculated that similar intracellular reactions occurred in the mucosal epithelium of oral leukoplakia and that p62 expression in the nucleus and p62 aggregation were increased in epithelial dysplasia lesions.

Recently, molecular biology approaches have been employed to clarify the mechanism of malignant transformation and identify several risk factors of malignancy as biomarkers, but there are only a few reports concerning autophagy‐related markers (Nikitakis et al., 2018).

Lévy et al. (2015) analyzed the levels of p62 to investigate the status of autophagy in intestinal epithelial cells during malignant transformation in *adenomatous polyposis coli*. They indicated that the inhibition of autophagy in the intestinal epithelium impaired the development and progression of tumors in patients with a high risk of tumorigenesis of colorectal cancer. Further, they indicated the important role of the microbiota in the antitumor effects of autophagy inhibition.

It has been shown that the ubiquitin/LC3‐binding protein p62 has a key role as a regulator of selective autophagy, as it is involved in both ubiquitinated antigen and LC3 (Komatsu et al., 2010; Ruan et al., 2017). Furthermore, it is necessary to sequester short‐lived proteins into the autophagosomes (Twitty, Jensen, Hu, & Fox, 2011). Terabe et al. (2018) reported that excessive accumulation of autophagy‐related proteins LC3A, LC3B, and p62 in normal mucosa near the surgical margin is a useful marker for local recurrence and associated poor prognosis. They clarified that the expression of LC3A and LC3B correlated with tumor recurrence and poor prognosis and the expression of p62 correlated with tumor recurrence. Therefore, they recommended that if these proteins are detected in normal mucosa near the surgical margin, strict follow‐up observation should be made or additional resection should be considered (Terabe et al., 2018). Otherwise, p62 can be considered as a target at the interface of aging, autophagy, and age‐related diseases (Bitto et al., 2014). p62 might be associated with various diseases, and it can be a potential biomarker of prognosis and survival of various malignancies induced by autophagy and oxidative stress.

The wild‐type p53 protein is degraded rapidly in cells, but the p53 mutant proteins possess an extended half‐life and accumulate in the cells. We can speculate that the presence of an abnormal *p53* gene results in the occurrence of the p53 mutant proteins (Hussain & Harris, 1998). The accumulation of p53 mutant proteins has been investigated, and frequent *p53* gene mutations are likely closely involved in the carcinogenesis of oral squamous cell carcinoma.(Sakai & Tsuchida, 1992) It is generally believed that p53 overexpression is significantly higher in oral leukoplakias that transform into cancer and that epithelial dysplasia is derived from p53‐mutated stem cells (Braakhuis, Leemans, & Brakenhoff, 2004). In the present study, an unknown causal relationship was observed between the nuclear p62 expression and p53 expression with epithelial dysplasia.

It is important to predict the possibility of malignant transformation and prognosis for various treatments of oral leukoplakia including surgery. Various causal factors for oral leukoplakia have been considered; these include smoking, excess alcohol drinking, betel quid chewing, and chronic infections like periodontitis. The mechanisms of malignant transformation have been investigated by many researcher; however, they have not been completely elucidated yet.

Therefore, several attempts are being made to identify specific molecular biomarkers to predict lesions that have the potential to transform into malignancy, as these criteria are beneficial to make a decision for the removal of lesions. There are a few reports that have demonstrated the significant association between nuclear p62 expression and p62 aggregation and epithelial dysplasia in oral leukoplakia.

Our results suggest that the intracellular expression of p62‐related biomarkers in the oral leukoplakia lesions has the potential to be developed as a tumor malignant transformation marker. Recently, aberrant intracellular expression of p62 has been identified in various solid tumors, including hepatocellular, breast, ovarian, prostate, oral, some gastrointestinal carcinomas, and melanoma (Ruan et al., 2017). Therefore, it has been considered that the inhibition of autophagy induces a marked cytoplasmic expression of p62 (Komatsu et al., 2007). Intracellular metabolism inhibition via p62 results in abnormal intracellular expression of genes, leading to tumorigenesis. A similar mechanism has been inferred in the malignant transformation of oral leukoplakia.

Due to the small sample size of this study, it is difficult to interpret the data for future clinical trials. We did not obtain quantitative data regarding each biomarker and immunohistochemistry was the only method used to evaluate p62 expression in human specimens (Ruan et al., 2017). However, to the best of our knowledge, there are no reports that indicate a significant association between p62 nuclear expression, p62 aggregation, and dysplasia in oral leukoplakia. In the present study, we showed that the expression of p62 is associated with dysplasia in oral leukoplakia. We have elucidated the clinical implementation of p62 as a biomarker to predict malignant transformation of oral leukoplakia. Nevertheless, we investigated only p62 expression in oral leukoplakia, but there are other autophagy‐related markers, such as LC3A/B, associated with poor prognosis in oral squamous cell carcinoma (Terabe et al., 2018). Further investigations are expected to clarify the malignant transformation mechanisms, elucidate the interactions among biomarkers, and identify new biomarkers.

In the future, well‐designed studies should investigate potential biomarkers to predict malignant transformation of oral leukoplakia to improve the treatment and diagnosis of oral premalignant lesions.

## CONFLICT OF INTEREST

The author(s) declare that there is no potential conflict of interest relevant to the study, authorship, and publication of this article.

## Supporting information

Figure S1. Box plot of positive cell occupancy rate of p62 expression in the nucleus, p62 aggregation, p62 expression in the cytoplasm, and 8‐OHdG, Ki67, and p53 expression.

Figure S2. Histogram of positive cell occupancy rate of p62 expression in the nucleus, p62 aggregation, p62 expression in cytoplasm, and 8‐OHdG, Ki67, and p53 expression.
