# Supplementary figures and images for: Association between p62 expression and clinicopathological characteristics in oral leukoplakia
Source: Clin Exp Dent Res. 2019 Jun 25;5(4):389–97. doi: 10.1002/cre2.193 (PMC6704027; doi:10.1002/cre2.193)

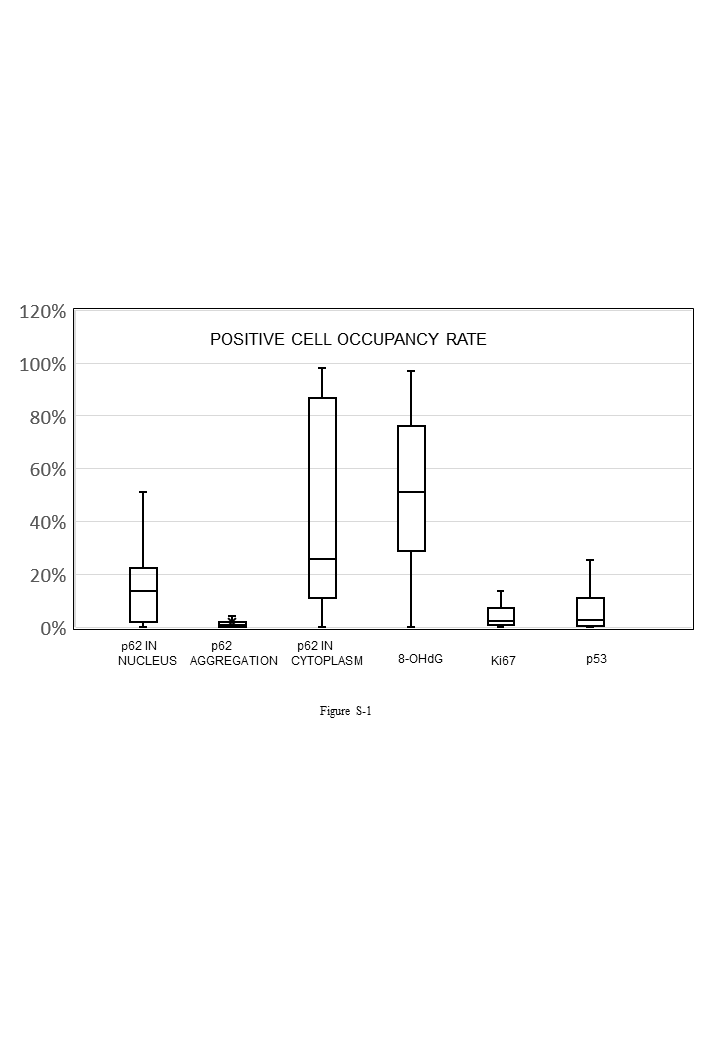

Supplement: Supplementary file 1 — Figure S1. Box plot of positive cell occupancy rate of p62 expression in the nucleus, p62 aggregation, p62 expression in the cytoplasm, and 8‐OHdG, Ki67, and p53 expression. [file CRE2-5-389-s001.TIF]

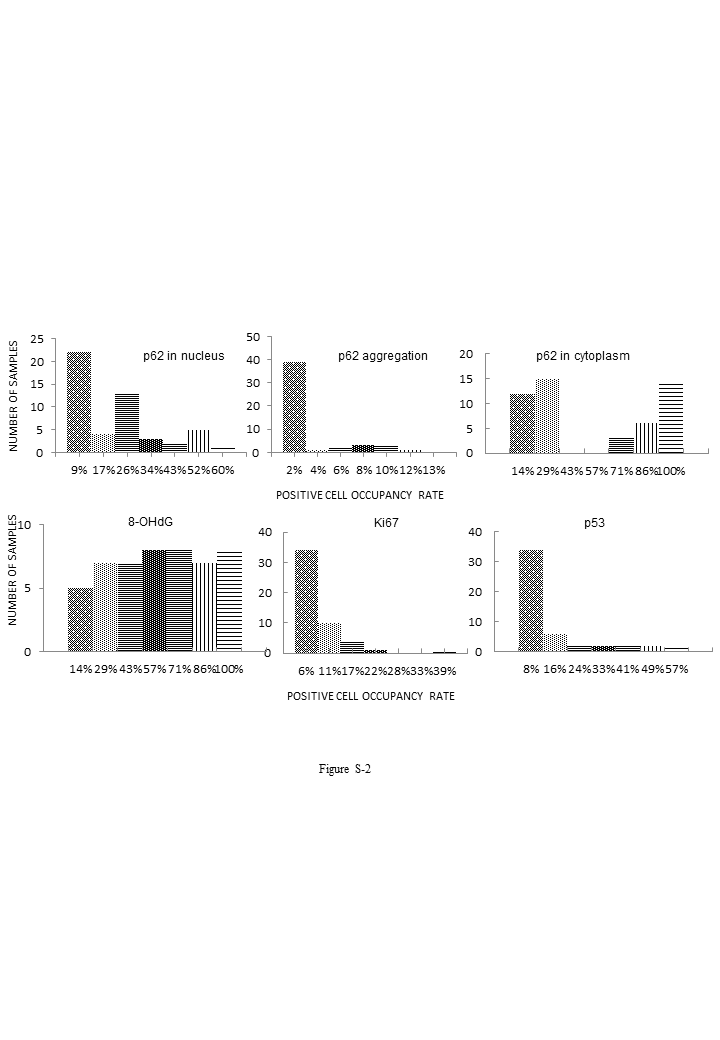

Supplement: Supplementary file 2 — Figure S2. Histogram of positive cell occupancy rate of p62 expression in the nucleus, p62 aggregation, p62 expression in cytoplasm, and 8‐OHdG, Ki67, and p53 expression. [file CRE2-5-389-s002.TIF]
